# Higher Socioeconomic Status is Associated with Improved Outcomes After Obesity Surgery Among Women in Germany

**DOI:** 10.1007/s00268-021-06252-8

**Published:** 2021-07-26

**Authors:** Jonas Wagner, Nicola Zanker, Anna Duprée, Oliver Mann, Jakob Izbicki, Stefan Wolter

**Affiliations:** Department of General-, Visceral- and Thoracic Surgery, University Medical Center Hamburg-Eppendorf, Martinistrasse 52, 20246 Hamburg, Germany

## Abstract

**Background:**

Low socioeconomic status (SES) is associated with an increased prevalence of obesity. It is unknown whether SES influences the outcome after bariatric surgery in Germany. Therefore, the aim of our study was to investigate whether the SES is linked with an inferior outcome after bariatric surgery.

**Methods:**

We included all patients who underwent bariatric surgery in our university hospital from 2012–2014. Net income was estimated by matching the zip codes of patient residency with the region-specific purchasing power index. We analyzed the relationship between SES, weight loss and remission of comorbidities.

**Results:**

We included 559 patients in this study and detected a mean 5-year percentage excess weight loss (%EWL) of 52.3%. We detected a significantly lower initial body mass index (BMI) and weight in patients with a higher income. One year after surgery, we did not find a significant difference. Further analysis revealed that only women with a higher income had a significantly lower BMI and weight 3 and 5 years after surgery.

**Conclusions:**

Bariatric surgery is beneficial for all patients regardless of income. Furthermore, we demonstrated that women with high SES have a better outcome after bariatric surgery.

## Introduction

The prevalence of obesity has doubled worldwide in recent years, making obesity one of the most important global challenges [1]. This also applies to Germany [2]. Obesity increases the risk of chronic diseases, including cardiovascular disease, diabetes mellitus, kidney diseases [3], musculoskeletal diseases [4] and cancer [5].

Bariatric surgery is important as the most successful treatment option for obesity [6]. Successful bariatric surgery is viewed as %EWL above 50% [7]. All current procedures are associated with sustained long-term weight loss [8]. Some factors are known to influence weight loss following surgery. The procedure type seems to affect the expected %EWL [8], while behavioral factors such as physical activity, dietary habits and health responsibilities do not affect the outcome [9].

Risk factors for developing obesity include the genetic predisposition, low physical activity, unhealthy eating behavior, sex and socioeconomic factors [10]. Socioeconomic factors are summarized as socioeconomic status, which is defined by the education, occupation and income of an individual or a group [11]. Obesity is distributed along a socioeconomic gradient, meaning that the prevalence of obesity is higher in people with low socioeconomic status [2]. Income alone influences the risk for obesity. This factor seems to be especially important for women [10].

A low SES is associated with a greater risk of chronic diseases and poorer outcome after therapy. Kucharska-Newton et al. showed that a low SES increases the risk of acute coronary heart disease events [12]. Additionally, Yong et al. reported that low SES leads to higher mortality after ST elevation myocardial infarction [13]. Regarding bariatric surgery, Carden et al. reported that in the USA, a lower SES is associated with reduced weight loss after bariatric surgery [14]. These aforementioned differences could partially be explained by accessibility of health care. In Germany, health insurance is mandatory, and healthcare access poses no problem [15]. Therefore, studying SES effects in Germany is a good choice, because the results should reflect true differences in SES, not in accessibility of health care.

However, the influence of SES on bariatric surgery in Germany has not been studied. It is relevant to explore whether SES, which influences the risk for obesity, also affects the outcome after bariatric surgery. Hence, we hypothesized that SES is in fact an influencing factor and is associated with an inferior outcome after bariatric surgery. Our study focused on the progression of weight, BMI, %TWL and %EWL after bariatric surgery with regard to SES. As a secondary outcome, remission of other weight-associated medical conditions was detected.

## Materials and methods

### Patient selection

We conducted a retrospective analysis of our database of patients, who underwent bariatric surgery between 2012 and 2014. Perioperative and follow-up data were available for 559 patients. Certified bariatric surgeons in a German bariatric Center of Excellence performed all surgical procedures. All patients were screened before surgery by a multidisciplinary team consisting of an endocrinologist, psychologist, nutritionist, physical therapist and surgeon. Patients were selected for surgery with a BMI > 40 kg/m^2^ or BMI > 35 kg/m^2^ and related comorbidities in accordance with the German Guidelines of Surgical Treatment of Obesity after discussion at our interdisciplinary obesity board. The operating surgeon decided which procedure to perform depending on BMI, comorbidities, medication and patient request [16]. Data regarding demographics, initial height, weight, BMI, comorbidities, procedure, HbA1c and length of follow-up, as well as the follow-up data after 1, 2, 3 and 5 years, including percent total weight loss (%TWL) and percent excess weight loss (%EWL), were collected. SES was indicated by net income per capita/month, which is an approved substitute marker. Income has previously been certified to correctly indicate SES [14, 17–20]. We used the purchasing power index (PPI) as a surrogate parameter to gain income information. The patient’s zip code was matched with the according PPI of the specific region, as has been used before [14, 21]. The PPI data were purchased from Michael Bauer Research GmbH. We used €2000 per capita/month as a cutoff based on the GEDA study [22].

The primary outcome was the total weight, BMI, %TWL and %EWL over time. Changes in other weight-associated medical conditions were examined as a secondary outcome. Patients under treatment regarding these conditions were defined as positive for weight-related diseases, according to the IDF consensus statement [23]. The American Society for Metabolic and bariatric Surgery criteria were used to define (partial) remission of weight-related diseases [24].

The local ethics committee approved the clinical database. All patients gave informed consent.

### Statistics

Statistical analysis was performed with the Statistical Package for Social Sciences software (SPSS; IBM, version 24). Patient characteristics are presented overall using the mean ± SD for continuous variables. For comparisons between continuous variables, independent Student’s t test was performed. Linear regression was performed to validate income/head/year as an independent variable. Multiple linear regression was computed to predict weight and BMI with sex, age, procedure, income/head and comorbidities as independent variables. To determine differences between nominal data, a Chi-square test was used and, if numbers of events were smaller than 5, Fisher’s exact test was performed. *p*-values < 0.05 were considered to be statistically significant.

## Results

### Patient’s characteristics

The characteristics of the patients are displayed in Table 1. A total of 559 patients were included in this study. Follow-up data were available for 66.7% of patients after 1 year, for 68.3% after 2 years, for 59.9% after 3 years and for 30.9% after 5 years. The initial mean weight was 149.8 ± 33.5 kg, with a BMI of 50.4 ± 9.9 kg/m^2^. The majority of the patients were female (69.4%). The average income/per capita/year was €24,128 ± 3469, while 54.2% had an estimated income of less than €24,000 annually. The most common comorbidity was hypertension (61.4%), followed by diabetes (33.6%) and OSA (13.4%) (Table 1).

**Table 1:** Patient characteristics

*n*	559
Age [years]	44.2 ± 11.5
Weight [kg]	149.8 ± 33.5
Height [cm]	172.2 ± 9.6
BMI [kg/m^2^]	50.4 ± 9.9
Women [n/%]	388/69.4
Men [n/%]	171/30.6
Procedure:	
Sleeve gastrectomy [n]	270
Gastric bypass [n]	260
Other^a^ [n]	29
Diabetes[n/%]	188/33.6
Hypertension[n/%]	343/61.4
OSAS[n/%]	75/13.4
Hypertriglyceridemia[n/%]	241/43.1
Hypercholesterolemia[n/%]	188/33.6
Average income [€/year]	24,128 ± 3469
Income < €24,000/year [n/%]	303/54.2

^a^Other procedures included gastric banding, single anastomosis duodeno-ileal bypass and conversion of sleeve gastrectomy to gastric bypass

### Overall time course of weight, BMI, %TWL and %EWL

**Fig. 1:**
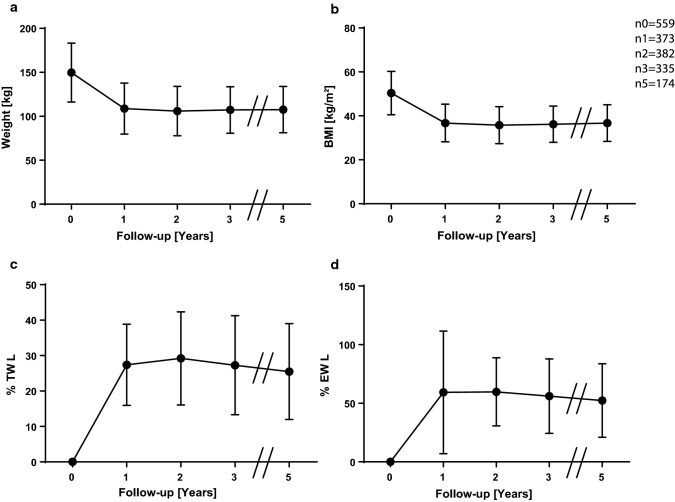
Means and standard deviations of weight **a**, BMI **b**, %TWL **c** and %EWL **d** of the general study population over 5 years. *n*_*t*_ is the number of available and analyzed patients, while _*t*_ is the timepoint in years postoperatively

We measured an average weight of 108.7 ± 29 kg of the cohort after 1 year. After 2 years, the weight reached its nadir with 106 ± 28 kg, then went slightly up after 3 years and remained stable after 5 years (Fig. 1a). The initial BMI dropped to 36.8 ± 8.5 kg/m^2^ after 1 year, resulting in a change of −13.6 kg/m^2^. The nadir was reached after 2 years, and the mean slightly increased after 3 years and stabilized after 5 years (Fig. 1b). After 1 year, we detected a %TWL of 27.4 ± 11.5%, which increased to 29.2 ± 13.1% after 2 years. In the following years, we observed a slight drop (Fig. 1c). The progression of %EWL was similar; after 1 year, it was 59.3 ± 52.3%, and then it reached its maximum 2 years postoperatively. It also dropped after 3 and 5 years postoperatively (Fig. 1d).

### Patients with higher income present themselves with lower weight and BMI

We divided the cohort into a low SES (*n* = 303) and a high SES group (*n* = 254). The low SES group was defined as an estimated income lower than €2000/month/per capita [22]. We observed an initial significantly lower weight and BMI in the high SES group (152.7 ± 35.4 kg vs. 146.2 ± 30.8 kg and 51.3 ± 10.3 kg/m^2^ vs. 49.2 ± 9.2 kg/m^2^) (*p* < 0.05). Additionally, the two groups only showed a significant difference in their average income (*p* < 0.05). Importantly, the groups were similar in procedure makeup (Table 2).

**Table 2:** Patient characteristics in terms of socioeconomic status

	Low SES (Income ≤ €24,000/year)	High SES (Income > €24,000/year)	*p* value
*n*	303	256	
Age [years]	43.7 ± 11.6	44.9 ± 11.4	0.22
Weight [kg]	152 ± 35	146 ± 30	0.023
Height [cm]	172.3 ± 9.6	172.1 ± 9.6	0.84
BMI [kg/m^2^]	51.3 ± 10.3	49.2 ± 9.2	0.016
Women [n/%]	209/69	179/70	0.8
Men [n/%]	94/31	77/30	0.8
Procedure:			0.17
Sleeve gastrectomy [n]	159	111	
Gastric bypass [n]	131	129	
Other^a^ [n]	13	16	
Diabetes[n/%]	109/36	79/31	0.2
Hypertension[n/%]	190/63	151/59	0.32
OSAS[n/%]	47/16	28/11	0.14
Hypertriglyceridemia[n/%]	123/41	118/46	0.12
Hypercholesterolemia[n/%]	105/35	82/32	0.64
Average income [€/year]	21,804 ± 1582	26,881 ± 3055	< 0.001

^a^Other procedures included gastric banding, single anastomosis duodeno-ileal bypass and conversion of sleeve gastrectomy to gastric bypass

**Fig. 2:**
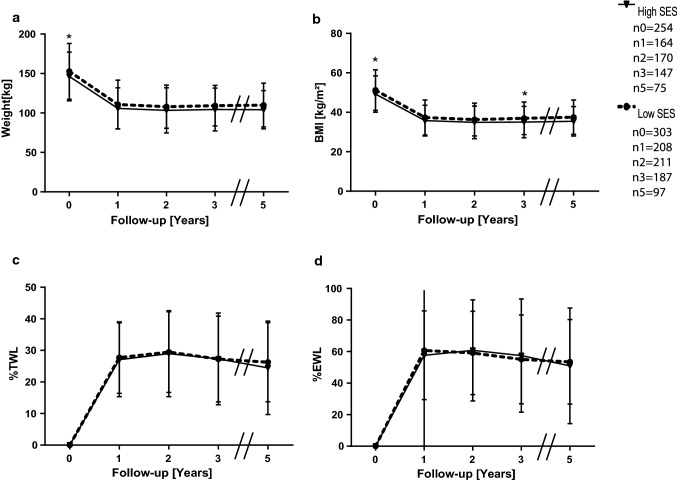
Means and standard deviations of weight **a**, BMI **b**, %TWL **c** and %EWL **d** of the study population over 5 years divided into their respective SES groups. n_t_ is the number of available and analyzed patients, while _t_ is the timepoint in years postoperatively. Follow-up rates for the high SES group were 64.6% after one year, 66.9% after two years, 57.9% after three years and 29.5% five years postoperative, for the low SES group the according rates were 68.6%, 69.6%, 61.7% and 32%. *p < 0.05

Over the course of the study period, the initial advantage vanished, although the high SES group had a significantly lower BMI after 3 years; after 5 years, no significant difference was detected (Fig. 2a, b). The course of the %TWL and %EWL showed no significant difference between the groups (Fig. 2c, d). We did not observe a difference in terms of remission of other weight-associated medical conditions (data not shown). We wondered whether we needed to reject our hypothesis that SES influences weight loss outcomes. Therefore, we performed linear regression analysis using income/head as an independent variable and BMI and weight as dependent variables. Although the proportion of explained variance was low, we observed that income was indeed an independent variable for BMI up to three years postoperatively and 1 year postoperatively for weight (Tables 3, 4).

**Table 3:** Linear regression of income/head on BMI.

BMI	1 year postoperative	2 years postoperative	3 years postoperative	5 years postoperative
Overall	0.024*	0.01*	0.013*	0.003
Male	0.025	0.001	0	0.012
Female	0.028*	0.02*	0.024*	0.005

Coefficient of determination (*R*^2^) as numerical value and significance (*)

^*^ denotes statistical significance (*p* < 0.05)

**Table 4:** Linear regression of income/head on weight.

Weight	1 year postoperative	2 years postoperative	3 years postoperative	5 years postoperative
Overall	0.016*	0.005	0.01	0.006
Male	0.022	0	0	0.005
Female	0.023*	0.022*	0.024*	0.012

Coefficient of determination (*R*^2^) as numerical value and significance (*)

^*^ denotes statistical significance (p < 0.05)

### Women benefit from a higher income regarding total weight and BMI

**Fig. 3:**
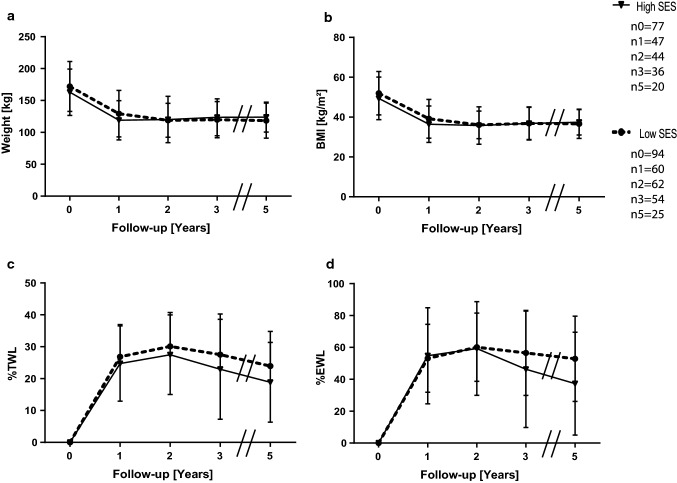
Means and standard deviations of weight **a**, BMI **b**, %TWL **c** and %EWL **d** for men in different SES classes. n_t_ is the number of available and analyzed patients for each group, while _t_ is the timepoint in years postoperatively. Follow-up rates for men in the high SES group were 61% after one, 57.1% after two, 46.8% after three and 25.9% five years postoperative, for the low SES group the according rates were 63.8%, 66%, 57.4% and 26.6%

**Fig. 4:**
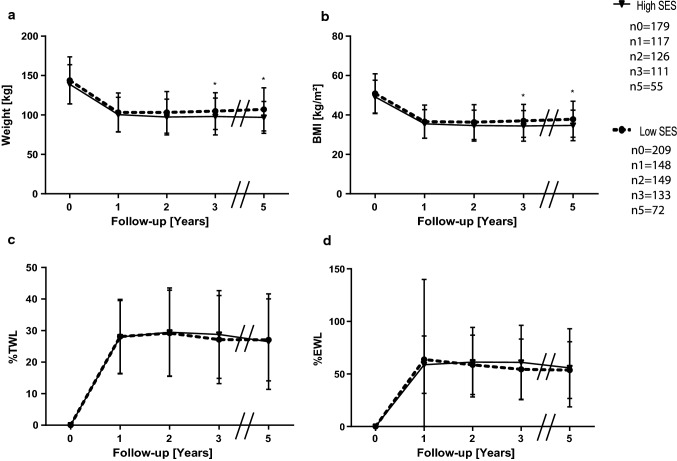
Means and standard deviations of weight **a**, BMI **b**, %TWL **c** and %EWL **d** for women in different SES classes. n_t_ is the number of available and analyzed patients for each group, while _t_ is the timepoint in years postoperatively. Follow-up rates for women in the high SES group were 65.3% after one, 70.3% after two, 62% after three and 30.7% five years postoperative, for the low SES group the according rates were 70.8%, 71.3%, 63.6% and 34.4%. *p < 0.05

We were looking for an explanation of why income was an independent variable for weight loss, but we did not see an effect in our data. Among females in highly developed countries such as Germany, low income is associated with higher weight [10, 25, 26]. Therefore, we analyzed the data in terms of sex differences. We did not find any significant differences in men with high SES (*n* = 77) compared to men with lower SES (*n* = 94) (Fig. 3a–d). The female groups presented themselves with no significant difference in weight or BMI prior to operation. After 3 years, women with high SES (*n* = 179) established a significantly lower weight (98 ± 23.3 kg vs. 104.9 ± 23.3 kg, *p* < 0.05) and BMI (34.5 ± 7.8 kg/m^2^ vs. 37 ± 8.4 kg/m^2^, *p* < 0.05) than females with low SES (*n* = 209). They also maintained a significant advantage in weight (97 ± 20.2 kg vs. 107 ± 27.3 kg) and BMI (34.7 ± 7.7 kg/m^2^ vs. 37.8 ± 9.1 kg/m^2^) 5 years postoperatively (Fig. 4a, b). The procedure makeup of both female groups was similar (Table 5), indicating that the observed effect was caused by differences in SES. Furthermore, no difference in %TWL, %EWL (Fig. 4c, d) or remission of other weight-associated medical conditions was observed (data not shown). We also performed linear regression and observed that income could only be regarded as an independent variable in females for BMI and weight up to three years postsurgery (Tables 3 and 4), further providing evidence that income affects only the weight loss of females postbariatric surgery.

**Table 5:** Female patient characteristics

	Low SES (Income ≤ €24,000/year)	High SES (Income > €24,000/year)	p value
n	209	179	
Age [years]	42.4 ± 10.7	43.5 ± 11.2	0.32
Weight [kg]	144 ± 29	139 ± 25	0.09
Height [cm]	168.1 ± 6.7	167.8 ± 6.8	0.74
BMI [kg/m^2^]	50.9 ± 10	49.3 ± 8.6	0.1
Procedure:			0.17
Sleeve gastrectomy [n]	95	65	
Gastric bypass [n]	105	107	
Other^a^ [n]	9	7	
Diabetes[n/%]	71/35	52/30	0.32
Hypertension[n/%]	125/60	96/54	0.2
OSAS[n/%]	24/14	21/13	0.91
Hypertriglyceridemia[n/%]	74/38	71/43	0.29
Hypercholesterolemia[n/%]	77/39.5	61/37.2	0.66
Average income [€/year]	21,830 ± 1526	26,656 ± 2631	< 0.001

^a^Other procedures included gastric banding, single anastomosis duodeno-ileal bypass and conversion of sleeve gastrectomy to gastric bypass

### Multiple linear regression analysis

Additionally, we performed multiple linear regression to see how income would perform compared to other variables. We used the type of procedure, income/head, comorbidities (diabetes, OSA, hypertension, dyslipidemia), sex and age as independent variables. This model showed a significant influence of the procedure type, income/head and diabetes on BMI one year after surgery. After two and three years, only the procedure was predictive. After 5 years, the procedure and OSA were predictive. We used the same variables to predict weight and observed a similar picture. One year after surgery, the type of procedure, income/head, sex and age significantly influenced the weight. After two and three years, only procedure and sex were predictive. After 5 years, we discovered the type of procedure, sex, OSA and dyslipidemia as influencing factors. We wondered whether these models would again perform better for female patients. The model for the female cohort showed a similar picture as the total cohort. The model could not predict BMI or weight for the male cohort (Tables 6, 7). This justified our sex-specific considerations further.

**Table 6:** Multiple linear regression for BMI. Adjusted coefficient of determination (R^2^) as numerical value, significance (*) and independent variables

**Weight**	1 year postoperative	2 years postoperative	3 years postoperative	5 years postoperative
Overall	0.126* ^1, 2, 3^	0.12* ^1^	0.105* ^1^	0.123* ^1, 6^
Male	NS	0.306* ^1, 9^	NS	0.366* (constant)
Female	0.149* ^1, 2^	0.114* ^1^	0.158* ^1, 6^	0.149* ^6, 9^

^*^ denotes statistical significance (p < 0.05), NS = not significant

Numbers denote significant variables (1 = procedure, 2 = income/head, 3 = diabetes, 4 = hypercholesterolemia, 5 = hyperglyceridemia, 6 = OSA, 7 = hypertension, 8 = sex, 9 = age)

**Table 7:** Multiple linear regression for weight. Adjusted coefficient of determination (R^2^) as numerical value, significance (*) and independent variables

Weight	1 year postoperative	2 years postoperative	3 years postoperative	5 years postoperative
Overall	0.234* ^1, 2, 8, 9^	0.244* ^1, 8^	0.211* ^1, 8^	0.29* ^1, 5, 6, 8^
Male	NS	0.284* ^1, 5^	NS	NS
Female	0.183* ^1, 2^	0.136* ^1, 2^	0.169* ^1^	0.149* ^1, 6^

^*^ denotes statistical significance (*p* < 0.05), NS = not significant

Numbers denote significant variables (1 = procedure, 2 = income/head, 3 = diabetes, 4 = hypercholesterolemia, 5 = hyperglyceridemia, 6 = OSA, 7 = hypertension, 8 = sex, 9 = age)

### Type of procedure

**Fig. 5:**
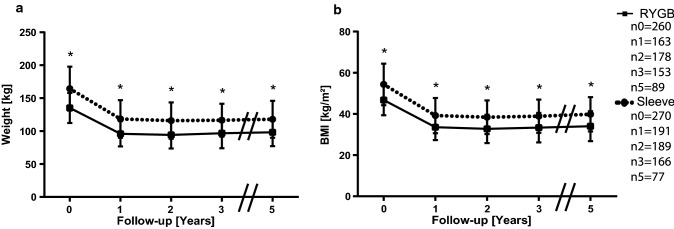
Means and standard deviations of weight **a**, BMI **b** for patients undergoing sleeve or bypass surgery. *n*_*t*_ is the number of available and analyzed patients for each group, while _*t*_ is the timepoint in years postoperatively. *p < 0.05

**Fig. 6:**
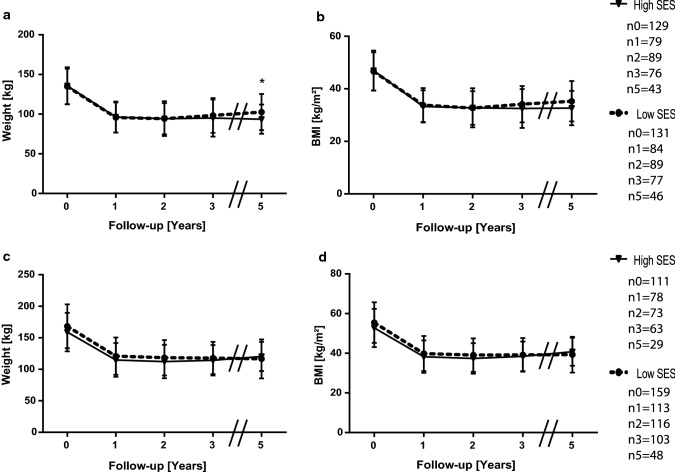
Means and standard deviations of weight **a**, **c**, BMI **b**, **d** for patients undergoing gastric bypass **a**, **b** or sleeve gastrectomy **c**, **d** in the different SES groups. n_t_ is the number of available and analyzed patients for each group, while _t_ is the timepoint in years postoperatively. *p < 0.05

However, the cohorts were homogenous regarding their type of procedures. We still wondered whether there was a procedure-specific effect. We compared the patients undergoing sleeve gastrectomy and patients undergoing gastric bypass surgery. Patients who received gastric bypass surgery had significantly (*p* < 0.05) lower BMI and weight (Fig. 5). Then, we split the cohorts based on income and procedure type. We observed significantly lower weight after 5 years in the high SES bypass group (Fig. 6a) but no difference in BMI (Fig. 6b). The sleeve groups only initially showed a significant difference (*p* < 0.05) in weight and BMI (Fig. 6c, d).

## Discussion

In this study, we assessed whether socioeconomic status impacts the outcome after bariatric surgery in Germany. First, we detected a good outcome after bariatric surgery, indicated by a %EWL above 50 through 5 years postoperatively, regardless of the SES. We observed that people with high SES present themselves with lower weight and BMI compared to patients with low SES. This difference could not be detected after 5 years. We used linear regression to show that income was indeed an independent variable for weight loss outcomes. In females, an inverse correlation exists between income and weight [10, 25, 26]. Therefore, we evaluated whether a sex-specific effect might be present. We observed that females with high SES had a lower weight and BMI up to 5 years postoperatively.

Previous studies that analyzed the impact of SES came to various conclusions. In 2008, Akkary et al. could not detect an advantage for patients with high SES [27]. More recently, Carden et al. reported that in a Veteran population, which consisted of mostly males, low SES led to significantly lower weight loss [14]. Our data suggest that only females with high SES have a better outcome. A study from Andersen et al. showed that predictors of weight loss following sleeve gastrectomy are sex-specific. Unemployment in women resulted in lower percent excess body mass index loss (%EBMIL) [28]. Which socioeconomic factors exactly influence the postoperative outcome remains to be examined. Women of lower-income classes have an increased risk for developing obesity [10]. Lower-income households seem to have less access to healthy food [29], and many women are still solely responsible for food purchase and preparation [25]. Access to physical activity or confidence to pursue physical activity can be reduced in women of low-income households [30]. Whether these factors also contribute to inferior weight loss in women with low SES after bariatric surgery remains to be determined. Further studies are needed to elucidate the exact socioeconomic aspects that contribute to a better outcome.

We observed that patients with high SES presented themselves with lower weight and BMI. Five years after surgery, this advantage disappeared. This indicates that bariatric surgery is distinguished regardless of SES. We detected no difference in %TWL and %EWL between the SES groups. The %TWL and %EWL are not the only markers for success after bariatric surgery and are not undisputed [31]. Individual patients are most likely only concerned about their total weight and BMI, since BMI is correlated with higher mortality [32]. This is the reason we focused on the total BMI and not the %TWL or %EWL.

This retrospective study suffers from some limitations. We used an approximation for the patient’s income, meaning that it is possible that patients were assigned to the wrong group. Additionally, the relocation of a patient could not be detected. However, this is an established tool [14, 21]. The strength of this study is the large sample size, which can reduce the errors of this tool. Another limitation is the low follow-up rate after 5 years (30.9%). Loss to follow-up is a problem after bariatric surgery, and attrition rates increase year by year [33]. However, through our large sample size, we still analyzed 174 patients and therefore concluded that our findings are valid.

Our results show that bariatric surgery is effective for all socioeconomic classes. Low income is a risk factor in women for an inferior outcome after bariatric surgery. Further studies should focus on elucidating the exact factors that induce these effects to aid all patients.

## Conflict of interest

The authors declare that they have no conflict of interest.

## Ethical approval

This study was approved by the local ethics committee.

## Informed consent

Informed consent was obtained from all individual participants included in the study.

## References

[1] Collaborators GO (2017). Health effects of overweight and obesity in 195 countries over 25 years. N Engl J Med.

[2] Hoebel J, Kuntz B, Kroll LE, Schienkiewitz A, Finger JD, Lange C, Lampert T (2019). Socioeconomic inequalities in the rise of adult obesity: a time-trend analysis of national examination data from Germany, 1990–2011. Obes Facts.

[3] Singh GM, Danaei G, Farzadfar F (2013). The age-specific quantitative effects of metabolic risk factors on cardiovascular diseases and diabetes: a pooled analysis. PLoS One.

[4] Jiang LTW, Wang Y, Rong J, Bao C, Liu Y, Zhao Y, Wang C (2012). Body Mass index and susceptibility to knee osteoarthritis: a systematic review and meta-analysis. Joint Bone Spine.

[5] Lauby-Secretan B, Scoccianti C, Loomis D, Grosse Y, Bianchini F, Straif K (2016). Body fatness and cancer—viewpoint of the IARC working group. New Engl J Med.

[6] Sjöström L, Narbro K, Sjöström D, Karason K, Larsson B, Wedel H, Lystig T, Sullivan M, Bouchard C, Carlsson B, Bengtsson C, Dahlgren S, Gummesson A, Jacobson P, Karlsson J, Lindroos AK, Lönroth H, Näslund I, Olbers T, Stenlöf K, Torgerson J, Ågren G, Carlsson LMS (2007). Effects of bariatric surgery on mortality in swedish obese subjects. New Engl J Med.

[7] Mann JP, Jakes AD, Hayden JD, Barth JH (2015) Systematic review of definitions of failure in revisional bariatric surgery. Obes Surg 25(3):571–574 10.1007/s11695-014-1541-2 25515500

[8] O'Brien PHA, Brennan L, Skinner S, Burton P, Smith A, Crosthwaite G, Brown W (2019). Long-term outcomes after bariatric surgery: a systematic review and meta-analysis of weight loss at 10 or more years for all bariatric procedures and a single-centre review of 20-year outcomes after adjustable gastric banding. Obes Surg.

[9] Ryder JR, Gross AC, Fox CK, Kaizer AM, Rudser KD, Jenkins TM, Ratcliff MB, Kelly AS, Kirk S, Siegel RM, Inge TH (2018). Factors associated with long-term weight-loss maintenance following bariatric surgery in adolescents with severe obesity. Int J Obes.

[10] Kuntz B, Lampert T (2010). Socioeconomic factors and obesity. Deutsches Ärzteblatt Int.

[11] Nakamura T, Nakamura Y, Saitoh S, Okamura T, Yanagita M, Yoshita K, Kita Y, Murakami Y, Yokomichi H, Nishi N, Okuda N, Kadato A, Ohkubo T, Ueshima H, Okayama A, Miura K (2018) Relationship between socioeconomic status and the prevalence of underweight, overweight or obesity in a general Japanese population: NIPPON DATA2010. J Epidemiol. 10.2188/jea.JE20170249 10.2188/jea.JE20170249 PMC5825685 29503379

[12] Kucharska-Newton AM, Harald K, Rosamond WD (2011). Socioeconomic indicators and the risk of acute coronary heart disease events: comparison of population-based data from the United States and Finland. Ann Epidemiol.

[13] Yong CM, Abnousi F, Asch SM (2014). Socioeconomic inequalities in quality of care and outcomes among patients with acute coronary syndrome in the modern era of drug eluting stents. J Am Heart Assoc.

[14] Carden A, Blum K, Arbaugh CJ (2019). Low socioeconomic status is associated with lower weight-loss outcomes 10-years after Roux-en-Y gastric bypass. Surg Endosc.

[15] Busse R, Blumel M (2014). Germany: Health system review. Health Syst Transit.

[16] Dietrich A, Aberle J, Wirth A (2018). Obesity surgery and the treatment of metabolic diseases. Dtsch Arztebl Int.

[17] van den Berg I, Buettner S, van den Braak R (2020). Low socioeconomic status is associated with worse outcomes after curative surgery for colorectal cancer: results from a large, multicenter study. J Gastrointest Surg.

[18] Lantz PM, House JS, Lepkowski JM (1998). Socioeconomic factors, health behaviors, and mortality: results from a nationally representative prospective study of US adults. JAMA.

[19] Sabanayagam C, Shankar A (2012). Income is a stronger predictor of mortality than education in a national sample of US adults. J Health Popul Nutr.

[20] Geyer S, Peter R (2000). Income, occupational position, qualification and health inequalities—competing risks?(Comparing indicators of social status). J Epidemiol Community Health.

[21] Roswall J, Almqvist-Tangen G, Holmén A, Alm B, Bergman S, Dahlgren J, Strömberg U (2016). Overweight at four years of age in a Swedish birth cohort: influence of neighbourhood-level purchasing power. BMC Public health.

[22] Lampert TKL, Müters S, Stolzenberg H (2013). Measurement of the socioeconomic status within the German Health Update 2009 (GEDA)Bundesgesundheitsblatt. Gesundh Gesundh.

[23] Alberti KG, Zimmet P, Shaw J (2006). Metabolic syndrome–a new world-wide definition. A consensus statement from the international diabetes Federation. Diabet Med J British Diabet Assoc.

[24] Brethauer SA, Kim J, el Chaar M (2015). Standardized outcomes reporting in metabolic and bariatric surgery. Surg Obes Relat Dis.

[25] McLaren L (2007). Socioeconomic status and obesity. Epidemiol Rev.

[26] Pavela G, Lewis DW, Locher J, Allison DB (2016). Socioeconomic status, risk of obesity, and the importance of Albert J. Stunkard Current. Obes Rep.

[27] Akkary E, Nerlinger A, Yu S (2009). Socioeconomic predictors of weight loss after laparoscopic Roux-Y gastric bypass. Surg Endosc.

[28] Andersen JR, Aadland E, Nilsen RM (2014). Predictors of weight loss are different in men and women after sleeve gastrectomy. Obes Surg.

[29] Lovasi GS, Hutson MA, Guerra M, Neckerman KM (2009). Built environments and obesity in disadvantaged populations. Epidemiol Rev.

[30] Bennet GG, McNeill LH, Wolin KY, Duncan DT, Puleo E, Emmons KM (2007). Safe to walk? Neighborhood safety and physical activity among public housing residents. PLoS Medicine.

[31] van de Laar A, de Caluwe L, Dillemans B (2011). Relative outcome measures for bariatric surgery. Evidence against excess weight loss and excess body mass index loss from a series of laparoscopic Roux-en-Y gastric bypass patients. Obes Surg.

[32] Prospective Studies C, Whitlock G, Lewington S (2009). Body-mass index and cause-specific mortality in 900 000 adults: collaborative analyses of 57 prospective studies. Lancet.

[33] O'Brien PE, Hindle A, Brennan L (2019). Long-term outcomes after bariatric surgery: a systematic review and meta-analysis of weight loss at 10 or more years for all bariatric procedures and a single-centre review of 20-year outcomes after adjustable gastric banding. Obes Surg.

